# Thrombus Aspiration in ThrOmbus containing culpRiT lesions in Non-ST-Elevation Myocardial Infarction (TATORT-NSTEMI): study protocol for a randomized controlled trial

**DOI:** 10.1186/1745-6215-14-110

**Published:** 2013-04-25

**Authors:** Suzanne de Waha, Ingo Eitel, Steffen Desch, Bruno Scheller, Michael Böhm, Bernward Lauer, Meinrad Gawaz, Tobias Geisler, Oliver Gunkel, Leonhard Bruch, Norbert Klein, Dietrich Pfeiffer, Gerhard Schuler, Uwe Zeymer, Holger Thiele

**Affiliations:** 1 Department of Internal Medicine/Cardiology, University of Leipzig - Heart Center, Struempellstr. 39, Leipzig 04289, Germany; 2 Department of Internal Medicine III, University of Saarland, Kirrberger Str. 100, Homburg, 06841, Germany; 3 Department of Cardiology, Zentralklinik Bad Berka, Robert-Koch-Allee 9, Bad Berka, 99437, Germany; 4 Department of Cardiology/Cardiovascular Medicine, University of Tuebingen, Otfried-Mueller-Str. 10, Tuebingen, 72076, Germany; 5 Department of Internal Medicine II, Klinikum Frankfurt/Oder, Muellroser Chaussee 7, Frankfurt/Oder, 15236, Germany; 6 Department of Internal Medicine, Unfallkrankenhaus Berlin, Warener Str. 7, Berlin, 12683, Germany; 7 Department of Internal Medicine I, University of Leipzig, Liebigstr. 20, Leipzig, 04103, Germany; 8 Institut für Herzinfarktforschung, Klinikum der Stadt Ludwigshafen, Bremserstr. 79, Ludwigshafen, 67063, Germany

**Keywords:** Non-ST-elevation myocardial infarction, Thrombectomy, Cardiac magnetic resonance imaging, Microvascular obstruction

## Abstract

**Background:**

Current guidelines recommend thrombus aspiration in patients with ST-elevation myocardial infarction (STEMI); however, there are insufficient data to unequivocally support thrombectomy in patients with non-STEMI (NSTEMI).

**Methods/Design:**

The TATORT-NSTEMI (Thrombus Aspiration in ThrOmbus containing culpRiT lesions in Non-ST-Elevation Myocardial Infarction) trial is a prospective, controlled, multicenter, randomized, open-label trial enrolling 460 patients. The hypothesis is that, against a background of early revascularization, adjunctive thrombectomy leads to less microvascular obstruction (MO) compared with conventional percutaneous coronary intervention (PCI) alone, as assessed by cardiac magnetic resonance imaging (CMR) in patients with NSTEMI. Patients will be randomized in a 1:1 fashion to one of the two treatment arms. The primary endpoint is the extent of late MO assessed by CMR. Secondary endpoints include early MO, infarct size, and myocardial salvage assessed by CMR as well as enzymatic infarct size and angiographic parameters, such as thrombolysis in myocardial infarction flow post-PCI and myocardial blush grade. Furthermore, clinical endpoints including death, myocardial re-infarction, target vessel revascularization, and new congestive heart failure will be recorded at 6 and 12 months. Safety will be assessed by the incidence of bleeding and stroke.

**Summary:**

The TATORT-NSTEMI trial has been designed to test the hypothesis that thrombectomy will improve myocardial perfusion in patients with NSTEMI and relevant thrombus burden in the culprit vessel reperfused by early PCI.

**Trial registration:**

The trial is registered under http://www.clinicaltrials.gov: NCT01612312.

## Background

Use of manual thrombectomy devices during percutaneous coronary intervention (PCI) in patients with acute ST-elevation myocardial infarction (STEMI) has been shown to decrease the incidence of cardiac death and repeat myocardial infarction [1-3]. Consequently, current guidelines of the European Society of Cardiology (ESC) and the American Heart Association/American College of Cardiology (AHA/ACC) recommend manual thrombectomy in patients with STEMI (class IIa, level of evidence A, and class IIa, level of evidence B, respectively) [4,5]. However, approximately 50 to 70% of all patients with non-ST-elevation myocardial infarction (NSTEMI) also display a relevant thrombus burden in the culprit vessel [6,7]. Furthermore, with rates of angiographic no-reflow phenomenon ranging from 15 to 40%, depending on thrombus burden and intra-procedural thrombotic events, only suboptimal reperfusion success can be achieved in a significant portion of patients with NSTEMI [8-10]. Thus, thrombectomy in patients with NSTEMI may represent a useful intervention, with the potential to improve patient prognosis. However, current guidelines do not give a clear recommendation for thrombus aspiration in NSTEMI [11,12]. This is mainly because of the lack of data on thrombus aspiration in NSTEMI, as only one small prospective observational study has been published to date [7]; in that 70-patient study, thrombectomy had a similar effectiveness rate to that reported in patients with STEMI.

This emphasizes the need for an adequately powered randomized controlled trial (RCT) to address the current role of thrombus aspiration in patients with NSTEMI and a relevant thrombus burden in the culprit vessel. The TATORT-NSTEMI (Thrombus Aspiration in ThrOmbus containing culpRiT lesions in Non-ST-Elevation Myocardial Infarction) trial is designed to test the hypothesis that against a background of early revascularization, adjunctive thrombectomy leads to less microvascular obstruction (MO) compared with conventional PCI alone, as assessed by cardiac magnetic resonance imaging (CMR).

## Methods/design

The TATORT-NSTEMI trial is a prospective, multicenter, randomized, controlled, open-label study to compare PCI plus routine thrombus aspiration with PCI without thrombus aspiration in patients with NSTEMI and relevant thrombus burden who are undergoing early invasive reperfusion therapy. The study will determine if adjunctive thrombectomy is superior than PCI alone with respect to the extent of no-reflow/MO assessed by CMR in patients with NSTEMI.

### Primary and secondary endpoints

The primary study endpoint of the TATORT-NSTEMI trial is the extent of late MO assessed by CMR on days 1 to 4 after randomization. Using CMR, zones of MO within the infarcted myocardium can be visualized and quantified, representing the CMR equivalent of the no-reflow phenomenon [13,14]. MO assessed by CMR has not only been shown to be associated with adverse functional outcome after myocardial infarction [15,16], but also with adverse clinical outcome including mortality [17-19]. Further, late MO assessed 15 minutes after contrast-medium injection has been shown to have higher prognostic impact than early MO assessed 1 to 2 minutes after gadolinium administration or by first-pass gadolinium-enhanced perfusion [17,20]. The mechanistic hypothesis of the current trial is that thrombus aspiration will result in reduction of distal embolization, thus preserving microvascular integrity, reflected by less late MO, which will translate into improved clinical outcome.

Secondary endpoints include other CMR parameters associated with patient prognosis, such as early MO, infarct size, myocardial salvage, and left ventricular ejection fraction (LVEF), as well as angiographic markers of reperfusion success such as the thrombolysis in myocardial infarction (TIMI) flow post-PCI and myocardial blush grade [18,21]. In addition, troponin T after 24 and 48 hours for determination of enzymatic infarct size will be evaluated. Further, a combined clinical endpoint including death, re-infarction, target vessel revascularization, and congestive heart failure (CHF) will be assessed after 6 and 12 months. Death is defined as death from any cause, but will be divided into cardiac and non-cardiac causes, and will be regarded as cardiac in origin unless obvious non-cardiac causes can be identified. The diagnosis of re-infarction during the index hospitalization will be based on clinical symptoms, new ST-segment changes, and an increase in the creatine kinase-MB levels above the reference limits in patients with normalized values, or if there is an increase of more than 20% from the most recent non-normalized measurement. At follow-up, any new ischemic symptoms leading to hospital admission accompanied by raised troponin levels will be defined as re-infarction [22]. New CHF after randomization during hospital stay will be defined as CHF when there is at least one of the following conditions and it requires treatment with diuretics (cardiogenic shock, pulmonary edema, or signs of pulmonary congestion on X-ray, rales of greater than one-third from lung basis, pulmonary capillary wedge pressure >25 mmHg, or dyspnea with blood oxygen saturation <90% in the absence of lung disease). After hospital discharge, any new CHF leading to re-hospitalization will be counted. Clinical outcome will be assessed by a telephone interview at 6 and 12 months. Any clinical event will be verified by hospital or general practitioner records. Finally, assessment of quality of life will be performed using the EuroQol-5D (http://www.euroqol.org) questionnaire during the 6-month and 12-month follow-ups.

Safety assessment will include stroke and bleeding until hospital discharge. Stroke will be defined as any new neurological symptoms in association with signs of ischemia or hemorrhage in cranial computed tomography or magnetic resonance imaging. Bleeding will be defined in accordance with the GUSTO (Global Utilization of Streptokinase and t-PA for Occluded Coronary Arteries) criteria as 1) severe or life-threatening, 2) moderate, or 3) mild bleeding [23]. However, based on previous studies, thrombus aspiration is associated with only modest complications, therefore no major safety concern for thrombus aspiration is expected. All safety aspects will be monitored by a Data Safety Monitoring Board (DSMB). All clinical and safety endpoints will be adjudicated by a Clinical Endpoints Committee (CEC) blinded to treatment assignment, based on data provided by the clinical-trial sites.

### Patient population

The study population will consist of 460 patients with NSTEMI enrolled at eight centers in Germany. Informed consent will be required prior to randomization.

Patients will be eligible for the study if they have: 1) ischemic symptoms such as angina pectoris for more than 20 minutes; 2) occurrence of previous symptoms less than 72 hours before randomization; 3) cardiac troponin levels above the 99th percentile; and 4) culprit lesion containing thrombus (TIMI thrombus grade 2 to 5 within the lesion) and intended early PCI.

Exclusion criteria will be presence of cardiogenic shock, STEMI, no identifiable culprit lesion, coronary morphology ineligible for thrombectomy (for example, very tortuous vessels or severe calcification), indication for acute bypass surgery, age less than 18 or more than 90 years, pregnancy, current participation in another clinical study, co-morbidity with limited life expectancy of less than 6 months or contraindications to CMR at study entry (for example, severe claustrophobia, implanted pacemakers or defibrillators, relevant metallic implants, known allergy to gadolinium, severe renal failure with creatinine clearance <30 ml/h), and contraindications for treatment with heparin, aspirin, or thienopyridines.

### Randomization and patient treatment

**Figure 1:**
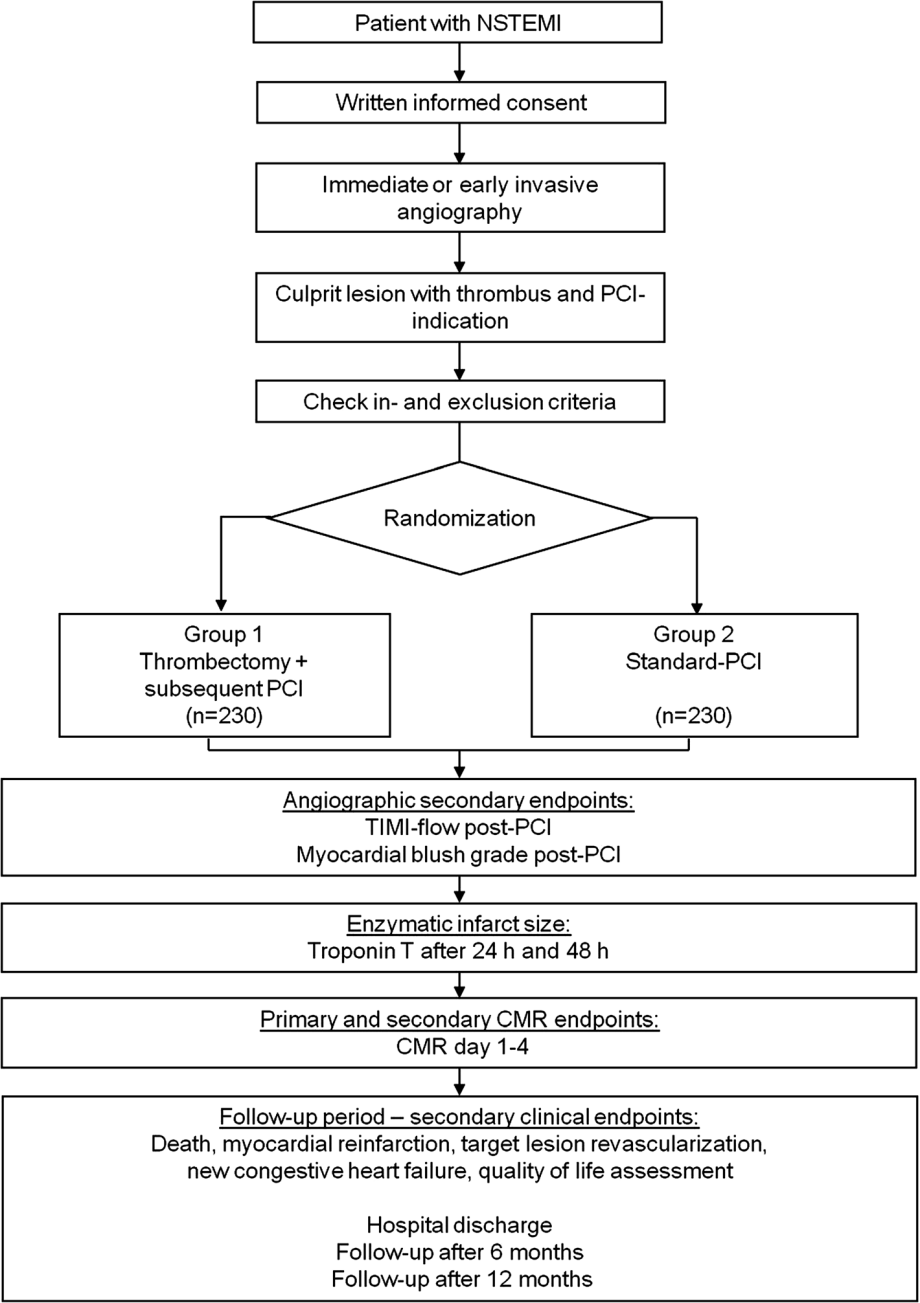
**Study flow chart. **CMR, cardiac magnetic resonance; NSTEMI, non-ST-elevation myocardial infarction; PCI, percutaneous coronary intervention; TIMI, thrombolysis in myocardial infarction.

Patient randomization will be performed centrally, with a randomization ratio of 1:1 (n = 230 patients per group; see Figure 1 for study flow) using an internet-based randomization program. No stratification will be performed. All participating hospitals are high-volume tertiary care centers with experienced cardiologists performing all interventions. Manual thrombectomy will be performed in the thrombus aspiration group with an aspiration catheter commonly used in daily clinical routine (Eliminate^®^; Terumo Europe, Leuven, Belgium). In the standard PCI group, patients will be treated by conventional PCI in accordance with local practice without thrombectomy. Cross-over is not planned; however, if this occurs, the reasons will be recorded. Manual thrombectomy will be the only difference between the groups, and all other therapeutic procedures will be similar in both treatment arms.

In vessels with a diameter of greater or equal than 2.5 mm, stent implantation is recommended. In both groups, the decision whether to use bare-metal or drug-eluting stents or whether to use glycoprotein IIb/IIIa inhibitors will be left to the discretion of the interventional cardiologist and the treatment decided upon will be recorded. Anticoagulant therapy including administration of heparin, bivalirudin, or low-molecular-weight heparin will be carried out in accordance with standard clinical practice and current guidelines. Additional treatment of other stenoses will be carried out if the interventional cardiologist deems it advisable, and will be recorded. Concomitant drug therapy for the treatment of NSTEMI will be administered based on the decision of the individual investigator and the common practice of the participating hospitals, in accordance with current guidelines. Anti-platelet therapy with initial loading dose will consist of aspirin 100 mg per day in combination with prasugrel 60 mg loading dose and subsequently 10 mg for 12 months for patients without contraindication to prasugrel. Patients pre-treated with clopidogrel should be switched to prasugrel whereas patients pre-treated with ticagrelor will not be switched. Post-infarction treatment includes administration of angiotensin-converting enzyme inhibitors or angiotensin-II-receptor antagonists, beta-blockers, and statins [11,12].

### Cardiac magnetic resonance image acquisition

**Figure 2:**
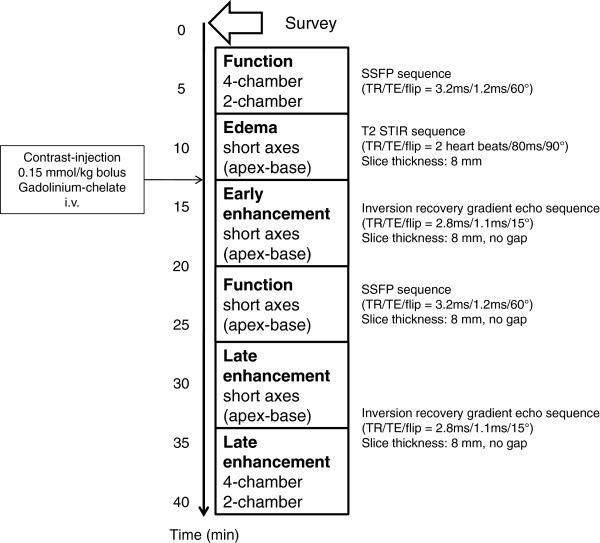
**Cardiac magnetic resonance imaging scan protocol. **Flip, flip angle; IR, inversion recovery; SSFP, steady-state free precession; STIR, short tau inversion recovery; repetition time; TE, echo time.

The primary endpoint (late MO) and secondary endpoints such as early MO, infarct size, and myocardial salvage will be assessed by CMR performed on a 1.5 or 3.0 Tesla scanner. Imaging will be performed on days 1 to 4 after randomization, using a standard scanning protocol (Figure 2). In brief, late MO and infarct size will be assessed in delayed enhancement short-axis images covering the whole ventricle acquired at mid-diastole approximately 15 minutes after bolus injection (0.2 mmol/kg bodyweight) of gadobutrol (Gadovist, Schering, Germany). An inversion recovery (IR) turbo gradient echo sequence will be used for image acquisition. The individual IR pre-pulse delay will be defined to obtain the maximal contrast between viable and necrotic myocardium. Early MO will be assessed 1 to 2 minutes after contrast-medium injection. For determination of edema/area at risk, short-axis slices covering the entire left ventricle (LV) before contrast administration will be obtained using a T2-weighted triple IR turbo spin-echo sequence. Assessment of LV function and volumes will be performed using a standard steady-state free precession technique, acquiring short-axis slices from base to apex and also horizontal and vertical long-axis views. Late and early MO, infarct size and area at risk will be expressed as percentages of the left ventricular mass (%LV), given by the sum of the mass of late and early MO regions, late enhancement and edema for all slices divided by the sum of the LV myocardial cross-sectional mass. The myocardial salvage index will be calculated as edema/area at risk minus infarct size divided by area at risk as described previously [24]. LVEF will be calculated from the short-axis functional views. The scan protocol and image analysis will be standardized at all sites and have been used effectively in previous studies [25-27]. All participating centers are experienced in using this type of conducted scan. CMR images will be sent to the CMR core laboratory at the University of Leipzig – Heart Center, Germany. The CMR core laboratory is highly experienced, has excellent reproducibility and low inter-observer and intra-observer variability for infarct size and myocardial salvage assessment, and has previously served as a core laboratory in studies of acute coronary syndromes [25-27]. Image analysis for the assessment of early and late MO, area at risk and infarct size, and total LV mass and volumes using a semi-automatic approach will be performed in anonymized form by blinded operators.

### Assessment of secondary angiographic endpoints

Both before and after PCI, the culprit vessel should be visualized in several projections in order to obtain particularly precise display of the distal segments. Evaluation of thrombus burden will be performed before and after PCI, in accordance with the TIMI thrombus grade scale.

The flow within the infarct-related artery will be assessed using the TIMI criteria and the corresponding myocardial blush grades [28,29]. For the assessment of myocardial blush, it is especially important for the investigators to provide film material with a sufficiently long duration to allow evaluation of myocardial perfusion. A central blinded analysis for TIMI flow, blush grade, and thrombus burden will be performed at the angiographic core lab of the University of Saarland.

### Biomarker sub-study

In a single-center sub-study (University Leipzig – Heart Center, Germany) blood will be collected from the femoral sheath (peripheral blood) and from the infarct-related coronary artery for biomarker sub-studies for approximately 200 patients. In this technique, the plasma is separated by centrifugation at 3500 *g* for 10 minutes, and aliquots are stored at -80°C until used. Multiple biomarkers such as markers of CHF, inflammation, or myocardial-derived microRNAs (miRNAs) will be analyzed. In addition, assessment of platelet function will be performed.

### Thrombus composition sub-study

In a two-center study (University of Leipzig - Heart Center and University of Tübingen, both in Germany) that will include approximately 100 patients, the composition and key determinants of aspirated thrombi will be analyzed. This will include histological and immunohistological analysis, and analysis of specific biomarkers such as thrombus miRNAs. The probes will be stored at -80°C and transferred to the core facility at the University of Tübingen.

### Data and statistical analysis

The aim of the study is to demonstrate superiority of manual thrombus aspiration versus standard PCI without thrombus aspiration, based on the primary endpoint of late MO in the study patients.

At the time of planning the study, the only available data on MO in NSTEMI were from a study on a small number (n = 25) of patients [30]. Thus, we initially based our sample-size calculation on previous in-house studies of the University of Leipzig - Heart Center in patients with STEMI and NSTEMI. An absolute difference in the primary endpoint (late MO) of 1.0%LV between the two treatment groups was expected, and a standard deviation of 3.3%LV was assumed [17,26,27,31]. Based on α = 5%, a two-sided hypothesis test, and a statistical power of 80%, 172 patients per group would be needed to reject the null hypothesis of no difference between groups.

In the intervening time, two other studies on MO and NSTEMI were published, which reported a lower prevalence and extent of MO than expected [20,32]. Thus, the Steering Committee decided to perform a preliminary analysis on CMR parameters, including the first 60 patients enrolled in TATORT-NSTEMI. A prevalence of MO of approximately 40% with a median extent of 1.0%LV was oberserved. We thus decided to critically revise our sample-size calculation and adapt it based on the new available data and the preliminary analysis. We now expect an absolute difference in the primary endpoint (late MO) of 0.5%LV between the two treatment groups with a mean extent of 1.0%LV for late MO in the whole study cohort [20,32]. This difference is judged as clinically relevant [17,20,26,27,31]. A standard deviation of 1.8%LV is assumed [17,20,26,27,31,32]. Based on α = 5%, a two-sided hypothesis test, and a statistical power of 80%, 204 patients per group are needed to reject the null hypothesis of no difference between groups. On the basis of clinical experience and data in STEMI, it is further expected that it will not be possible to perform thrombectomy for 5% of all patients in the thrombectomy group, and that 10% of all patients, either will not undergo a CMR examination or their CMR images will be of insufficient quality for analysis [17,26,27]. This will result in a sample size of 2 × 230 randomized patients per group. We thus decided to include 60 additional patients after critical revision and adaptation of our initial sample-size calculation. This protocol modification was approved by the central ethics committee at the University of Leipzig. Sample-size calculation was performed using SigmaStat software (Version 3.5; Systat Software Inc., Chicago, IL, USA).

Pre-specified subgroup analyses by gender, presence of diabetes, TIMI thrombus burden (2 to 4 versus 5), TIMI flow pre-PCI (0 to 1 versus 2 to 3), and time from most recent symptoms to occurrence of PCI (<6 versus ≥6 hours), and additional administration of glycoprotein IIb/IIIa inhibitors will be performed.

All patients will be analyzed on an intention-to-treat basis. The differences in primary and secondary endpoints between the treatment groups will be assessed using an unpaired *t*-test. In addition, as a secondary analysis, the differences between the treatment groups in the main outcome will be assessed using an unpaired *t*-test adjusted by baseline values (ANCOVA). Categorical variables will be analyzed by Fisher’s exact test. The 95% confidence interval (CI) for differences in quantitative data will be displayed. For clinical events, relative risks and 95% CI will be reported. To identify factors associated with the observed results, univariate and multivariate regression analysis will be performed. To assess clinical outcome, Kaplan-Meier curves with log-rank comparison and Cox regression analyses will be performed. A two-tailed *P*-value of <0.05 will be considered significant.

### Study organization

The TATORT-NSTEMI Steering Committee is chaired by Prof. Dr. Holger Thiele (University of Leipzig – Heart Center, Leipzig, Germany), co-chaired by Prof. Dr. Uwe Zeymer, (Institut für Herzinfarktforschung [IHF], Ludwigshafen, Germany), and Prof. Dr. Bruno Scheller (University of Saarland, Campus Homburg/Saar, Homburg, Germany). The Steering Committee is responsible for the scientific content of the protocol and oversees the trial operations, and will perform the preparation of the primary manuscript and other publications arising from the TATORT-NSTEMI trial. Each participating center has a coordinator with extensive clinical-trial experience. All trial-related processes will follow the standard operating procedures of the IHF, which is an independent clinical-research organization providing biometry, study coordination, monitoring, and data management. Central monitoring will include a timely query-management process based on consistency and plausibility checks automatically generated from the database, combined with a dunning process for missing documentation and a reminder system in advance for upcoming visits. On-site monitoring will include initiation, regular visits, and close-out. During recruitment and follow-up, all centers will be visited regularly to check adherence to good clinical practice and trial protocol. The focus of the visits will be on the verification of informed-consent documents, eligibility criteria, key primary and secondary endpoints, and safety aspects.

The trial will be monitored by an independent DSMB. The CEC will adjudicate all clinical endpoints. The trial follows a standard study organization with a Steering Committee, DSMB, and CEC.

The study has been approved by the central ethical committee at the University of Leipzig and at all local ethics committees at the participating sites.

## Discussion

Despite early invasive strategies and optimal medical treatment, morbidity and mortality rates for NSTEMI and STEMI continue to be high. Thus, further improvement of the initial therapy is necessary to improve patient outcome. The pivotal TAPAS (Thrombus Aspiration during Percutaneous coronary intervention in Acute myocardial infarction Study) trial of patients with STEMI, which compared a strategy with thrombectomy with a strategy without thrombectomy, showed a reduction in cardiac mortality at 1 year [1]. By contrast, other randomized, controlled studies did not observe superiority of thrombectomy over standard PCI with respect to surrogate endpoints of reperfusion success [33,34]. However, meta-analyses in patients with STEMI showed a mortality-related benefit after thrombectomy compared with PCI alone [2,3]. Therefore, the current guidelines of the ESC on revascularization therapy in patients with STEMI reperfused by primary PCI have increased the recommendation class for manual thrombectomy from the previous class IIb, level of evidence B to class IIa, level of evidence A [4]. In line with the ESC guidelines, thrombectomy is strongly recommended by the AHA/ACC guidelines (class IIa, level of evidence B) [5].

Notably, in the majority of both STEMI and NSTEMI cases, the key pathophysiological substrate is rupture of a vulnerable plaque with subsequent coronary thrombosis. Whereas in STEMI the thrombus is mostly fibrin-rich leading to total vessel occlusion, the thrombus in many patients with NSTEMI is predominantly platelet-rich and unstable. leading to partial or intermittent occlusion of the coronary vessel [35]. The majority of patients with NSTEMI display relevant thrombus burden and thus might benefit from thrombectomy [6,7]; however, current guidelines do not recommend thrombus aspiration in patients with NSTEMI [11,12]. This is mainly because of the lack of data for patients with NSTEMI, with only one published prospective observational study to date [7]. In that study, which enrolled 70 patients with NSTEMI, a thrombus was detected by the initial angiogram in approximately 50% of participants. No complications occurred during thrombectomy. Manual thrombus aspiration was associated with a significant reduction of the TIMI thrombus score (score 4/5 in 40% of patients before thrombectomy versus 7% after thrombectomy) and an increase in the rate of TIMI flow 3 (in 36% pre-thrombectomy versus 66% post-thrombectomy). After thrombus aspiration, direct stenting without pre-dilatation was possible in 39 patients (55.7%). Thus, this study showed that thrombus aspiration in patients with NSTEMI is feasible, effective, and safe.

However, RCTs are necessary to investigate whether thrombus aspiration in NSTEMI results in improved angiographic, functional, and clinical outcomes compared with conventional PCI. Currently, one additional randomized single-center study comparing thrombectomy with PCI alone is ongoing (TAPAS-II study) [36]. The primary endpoint of that study is myocardial blush grade, and the study aims to enroll 560 patients with NSTEMI. The main limitations of that study are its single-center design and the performance of unselected thrombectomy in all patients, independent of thrombus presence. In addition, the primary endpoint of myocardial blush grade is only an indirect marker for myocardial perfusion. By contrast, the primary endpoint of our proposed study, late MO by CMR, allows direct visualization and quantification of no-reflow and microcirculatory impairment [15,16]. Both myocardial blush and MO are surrogate endpoints, and evidently assessing clinical outcome as primary endpoint would lead to more robust data. However, late MO has been shown to be a strong independent predictor for clinical outcome, including mortality, after acute coronary syndromes [17-19]. The main pathophysiological mechanism for the development of MO is distal embolization [13,14]. Because manual thrombus removal reduces thrombus burden and thereby distal embolization, the primary endpoint of the current trial (MO) and the studied intervention (manual thrombectomy) are directly and causally linked with each other on a pathophysiological basis.

Previous data for patients with STEMI indicate that in approximately 10% of all patients the thrombus aspiration catheter fails to cross the lesion [37]. Because lesions in NSTEMI are often less complex and frequently display lower-grade stenoses, it can be expected that it will not be possible to perform thrombectomy in a smaller percentage (approximately 5%) of all patients in the thrombectomy group. To reduce the rate of unsuccessful thrombectomy, presence of a coronary morphology ineligible for thrombectomy (for example, patients with very tortuous vessels or severe calcification) will be a primary exclusion criterion. In addition, only interventional cardiologists experienced in manual thrombectomy will participate in this trial. Finally, blinding will not be possible as a result of the intervention used. However, several methods to restrict bias will be implemented, such as a central computerized randomization system; a blinded CEC to assess all relevant clinical events; a blinded CMR and angiographic core laboratory; and high standard requirements concerning the clinical level and experience of centers and investigators in terms of numbers of PCIs performed, cases of NSTEMI treated, and participation in cardiologic clinical trials.

In clinical trials using CMR parameters as surrogate endpoints, the time period for CMR image acquisition after the index event or randomization is of importance for the final quality and reproducibility of the results. However, previous studies analyzing the influence of timing of CMR image acquisition on CMR parameters led to inconsistent results. In contrast to previously published animal data with stable infarct size and MO measurements between day 2 and 7 after the index event [38],there has been increasing evidence recently based on findings in humans that infarct size decreases within the first week after the initial event [39-42]. However, these studies included only small study sample sizes, and to date, the precise devolution of the infarcted areal within the first days has not been investigated. Thus, based on current data and practicability of performance of CMR within clinical routine times to assure high-quality image acquisition, we chose to acquire CMR images within the narrow time period of 1 to 4 days after randomization. Furthermore, we do not expect differences in timing of CMR in both treatment groups. Thus, even if there were to be an increase or decrease in MO over time in humans, this would not affect the study results.

## Trial status

Recruitment began in March 2011 and of December 2012, almost 360 patients had been enrolled. Given these inclusion rates, completion of enrollment is expected by July 2013. It is expected that complete data will be available in September 2013.

## Abbreviations

AHA/ACC: American Heart Association/American College of Cardiology; CEC: Clinical Endpoints Committee; CHF: congestive heart failure; CI: Confidence interval; CMR: Cardiac magnetic resonance; DSMB: Data Safety Monitoring Board; ESC: European Society of Cardiology; IHF: Institut für Herzinfarktforschung; LV: Left ventricle; LVEF: Left ventricular ejection fraction; MO: Microvascular obstruction; NSTEMI: Non-ST-elevation myocardial infarction; PCI: Percutaneous coronary intervention; RCT: Randomized controlled trial (RCT); STEMI: ST-elevation myocardial infarction; TATORT-NSTEMI: Thrombus Aspiration in ThrOmbus containing culpRiT lesions in Non-ST-Elevation Myocardial Infarction; TIMI: Thrombolysis in myocardial infarction.

## Competing interests

The authors do not have competing interests concerning the TATORT-NSTEMI trial.

## Authors’ contributions

All authors have made substantial contributions to conception and design of the current trial and have been involved in drafting the manuscript or revising it critically for important intellectual content. All authors have given final approval of the manuscript.
